# The effect of DPP-4 inhibition to improve functional outcome after stroke is mediated by the SDF-1α/CXCR4 pathway

**DOI:** 10.1186/s12933-018-0702-3

**Published:** 2018-05-19

**Authors:** Fausto Chiazza, Harald Tammen, Hiranya Pintana, Grazyna Lietzau, Massimo Collino, Thomas Nyström, Thomas Klein, Vladimer Darsalia, Cesare Patrone

**Affiliations:** 1 Department of Clinical Science and Education, Södersjukhuset, Internal Medicine, Karolinska Institutet, 118 83 Stockholm, Sweden; 2 Department of CardioMetabolic Diseases Research, Boehringer Ingelheim Pharma GmbH & Co. KG, Biberach, Germany; 3 Department of Drug Science and Technology, University of Turin, Torino, Italy; 4 PXBioVision GmbH, Hannover, Germany

**Keywords:** Gliptins, DPP-4 inhibitors, Linagliptin, CXCR4/SDF-1α, Stroke, MCAO, Diabetes

## Abstract

**Background:**

Dipeptidyl peptidase-4 (DPP-4) inhibitors (gliptins) are approved drugs for the treatment of hyperglycemia in patients with type 2 diabetes. These effects are mainly mediated by inhibiting endogenous glucagon-like peptide-1 (GLP-1) cleavage. Interestingly, gliptins can also improve stroke outcome in rodents independently from GLP1. However, the underlying mechanisms are unknown. Stromal cell-derived factor-1α (SDF-1α) is a DPP-4 substrate and CXCR4 agonist promoting beneficial effects in injured brains. However, SDF-1α involvement in gliptin-mediated neuroprotection after ischemic injury is unproven. We aimed to determine whether the gliptin linagliptin improves stroke outcome via the SDF-1α/CXCR4 pathway, and identify additional effectors behind the efficacy.

**Methods:**

Mice were subjected to stroke by transient middle cerebral artery occlusion (MCAO). linagliptin was administered for 3 days or 3 weeks from stroke onset. The CXCR4-antagonist AMD3100 was administered 1 day before MCAO until 3 days thereafter. Stroke outcome was assessed by measuring upper-limb function, infarct volume and neuronal survival. The plasma and brain levels of active GLP-1, GIP and SDF-1α were quantified by ELISA. To identify additional gliptin-mediated molecular effectors, brain samples were analyzed by mass spectrometry.

**Results:**

Linagliptin specifically increased active SDF-1α but not glucose-dependent insulinotropic peptide (GIP) or GLP-1 brain levels. Blocking of SDF-1α/CXCR4 pathway abolished the positive effects of linagliptin on upper-limb function and histological outcome after stroke. Moreover, linagliptin treatment after stroke decreased the presence of peptides derived from neurogranin and from an isoform of the myelin basic protein.

**Conclusions:**

We showed that linagliptin improves functional stroke outcome in a SDF-1α/CXCR4-dependent manner. Considering that Calpain activity and intracellular Ca^2+^ regulate neurogranin and myelin basic protein detection, our data suggest a gliptin-mediated neuroprotective mechanism via the SDF-1α/CXCR4 pathway that could involve the regulation of Ca^2+^ homeostasis and the reduction of Calpain activity. These results provide new insights into restorative gliptin-mediated effects against stroke.

**Electronic supplementary material:**

The online version of this article (10.1186/s12933-018-0702-3) contains supplementary material, which is available to authorized users.

## Background

Dipeptidyl-peptidase 4 (DPP-4) inhibitors (gliptins) are oral antidiabetic drugs used to treat type 2 diabetes mellitus (T2D). Gliptins mediate their anti-diabetic effects by primarily inhibiting degradation of endogenous glucagon-like peptide 1 (GLP-1) and glucose-dependent insulinotropic peptide (GIP), resulting in prolongation of postprandial insulin secretion [1]. Recent research has shown that gliptins can also reduce stroke-induced brain damage in animal models in presence or absence of diabetes [2]. Furthermore, several reports have shown that gliptins mediate positive pleiotropic effects in animal models of Alzheimer’s disease (AD) [3–7] and in diabetic patients with AD [8]. Translation of these positive functional results to diabetic (and non-diabetic) individuals affected by stroke remains to be demonstrated since large clinical studies have not yet evaluated the potential of gliptins in improving functional stroke outcomes [9]. Instead, these studies assessed gliptins’ efficacy to prevent cardiovascular events (including stroke) and to decrease mortality in people with diabetes with basically neutral results [2, 10, 11].

The molecular mechanisms underlying gliptin-mediated effects in brains are also largely unknown. GLP-1 and GIP are regarded as main DPP-4 substrates. However, we recently showed that Linagliptin can improve stroke outcome independently from glycemia regulation [12] and GLP-1R [13]. These data indicate that one or more additional DPP-4 substrates with direct or indirect neuroprotective properties may be involved in gliptin-mediated brain effects. GIP can play a role in neuroprotection after stroke [14]. However, DPP-4 also cleaves other peptides, of which many exhibit direct actions on the cardiovascular system [15–17]. Among these, a promising candidate is the C-X-C motif chemokine 12 (CXCL12) [stromal cell-derived factor 1 alpha (SDF-1α)], which has been demonstrated to be fundamentally involved in brain homeostasis [18]. SDF-1α is a small cytokine mediating mobilization and homing of bone marrow-derived stem and progenitor cells in vascular injury [19], lymphopoiesis, myelopoiesis and germ cell mobilization [20]. To exert its actions, SDF-1α activates two receptors, CXCR4 and CXCR7 [19]. SDF-1α and its receptor CXCR4 are abundant and ubiquitously expressed in the developing and mature central nervous system, playing a role in neurogenesis and contributing to the neuronal development [19, 21]. Furthermore, the levels of SDF-1α and expression of CXCR4 in plasma and cerebrospinal fluid were decreased in clinical and preclinical studies of AD and negatively correlated to changes in cognitive functions [22]. The role of SDF-1α in cerebral ischemic injury is complex since some studies have shown positive effects of SDF-1α in the acute phase after stroke [23, 24] whereas other studies have demonstrated positive effects by blocking the SDF-1α/CXCR4 pathway in the recovery phase after stroke [25, 26].

In the present study, we investigated whether protective effects of linagliptin after stroke are mediated via SDF-1α by blocking CXCR4 with the selective CXCR4 antagonist AMD3100. Additionally, by using tandem mass spectrometry, we identified effectors putatively involved in gliptin-mediated effects.

## Methods

### Animals and experimental groups

Animals were housed on a 12 h light/dark cycle with ad libitum access to food/water. Experiments were conducted in accordance with the Guidelines for Care and Use of Laboratory Animals published by US National Institute of Health (Eighth edition, 2011).

101 adult male C57bl6/j mice were used in 4 studies.

#### Study 1 (to establish an experimental setting to study the effects of linagliptin [27] on functional and histological outcomes after stroke)

To determine whether linagliptin reduces ischemic tissue damage and improves functional outcome when given after experimental stroke, 16 mice were subjected to transient middle cerebral artery occlusion (MCAO, see below) and treated with linagliptin (n = 9) or vehicle (natrosol n = 7). The first week, linagliptin was administered once daily *per oral gavage* at 10 mg/kg/bw beginning the day of stroke onset. During the next 2 weeks, to diminish *per oral gavage*-induced stress, mice received linagliptin mixed with standard laboratory chow at a concentration of 83 mg linagliptin pro kg chow. At this concentration, the plasma levels of linagliptin reach approximately 50–100 nM [28], equal to an estimated daily intake of 5–8 mg/kg/bw. Both regimes of administration were efficacious in the brain in previous studies [12, 13, 29, 30]. Mice were killed 3 weeks after MCAO. Motor function (see below) was assessed before, 3 days and 3 weeks after MCAO.

#### Study 2 (to quantify active GIP, GLP-1 and SDF-1α after sustained linagliptin treatment)

To determine whether linagliptin treatment up-regulates active GLP-1, active GIP and active SDF-1α in the brain, 19 mice were given linagliptin (n = 10, following the same administration protocol of study 1) or vehicle (natrosol n = 9) daily. Mice were killed after 3 weeks and brain tissues were isolated. Active GLP-1, active GIP and active SDF-1α were determined in serum and brain tissues using an enzyme-linked immunosorbent assay (ELISA; see below).

#### Study 3 (to determine the potential role of the SDF-1α/CXCR4 pathway in linagliptin-mediated efficacy in the acute phase after stroke)

To determine whether the improved stroke outcome after linagliptin treatment was mediated by the SDF-1α/CXCR4 pathway, 58 mice were subjected to MCAO and treated with linagliptin (10 mg/kg/bw, *per oral*, n = 19), AMD3100 (5 mg/kg/bw, *intraperitoneal* n = 11; [31]) AMD3100 + linagliptin (n = 11) or vehicle (natrosol n = 17) for 3 days and sacrificed thereafter. AMD3100 is a strong, almost irreversible antagonist of CXCR4 [32]. To maximally block the effect of CXCR4 on the acute phase after stroke, AMD3100 was given starting from 1 day before MCAO. Subsequently, immunohistological measurements of ischemic brain damage were performed. All animals were tested for motor function before and 3 days after MCAO.

#### Study 4 (to identify linagliptin-mediated effectors in the acute phase after stroke by mass spectrometry)

For mass spectrometric analysis, 8 mice subjected to stroke from Study 3 and treated with linagliptin (10 mg/kg/bw, *per oral*, n = 5) or vehicle (n = 3) were employed. Additional 8 naive mice were treated with linagliptin (10 mg/kg/bw, *per oral*, n = 4) or vehicle (n = 4). All mice were sacrificed after 3 days. Brain tissues were isolated, frozen and analyzed by mass spectrometry.

A representative illustration of study design is provided in Additional file 1: Figure S1.

### Middle cerebral artery occlusion (MCAO)

The intraluminal filament technique was used [33]. Briefly, animals were anesthetized using 1.5% isoflurane, carotid arteries on the left side were exposed, the external carotid was ligated and temporary sutures were placed over the common carotid artery. Through a small incision in the external carotid artery, a 7–0 monofilament coated with silicone was advanced through the internal carotid artery until it blocked the origin of the middle cerebral artery. When the filament had been positioned, wounds were closed and the anesthesia was discontinued. After 30 min of occlusion, the mice were anesthetized again, the filament was withdrawn and ligatures removed from the common carotid artery. Body temperature was maintained at 37–38 °C with a heated pad during surgery. The mice were then transferred to a heated box where they regained wakefulness and were kept there for 2 h.

### Assessment of motor function

Motor performance was assessed using forepaw grip strength test [34] before MCAO, 3 days and 3 weeks after MCAO. Briefly, animals were allowed to grasp the handlebar connected to a force meter and gently dragged backward until the grip was released. 10 trials were performed and the highest values recorded. Grip strength of left and right forepaws was measured separately and motor asymmetry was determined by the left to right forepaw strength ratio. The left to right forepaw strength ratio for each mouse before MCAO was measured and used for normalization for post MCAO ratios.

### Immunohistochemistry

Animals were deeply anesthetized with sodium pentobarbital and transcardially perfused with saline followed by 4% ice-cold paraformaldehyde. Brains were extracted, post-fixed in 4% paraformaldehyde at 4 °C overnight and submersed in 20% sucrose in phosphate buffer until they sank. 50 µm-thick coronal sections were cut using a sliding microtome and stained as free-floating sections. The details of the immunohistochemistry, and infarct volume and cell quantifications have been recently described [12, 13] and are provided in Additional file 1.

All tests and procedures were performed by an experimenter blinded to the experimental groups.

### Determination of active GLP-1, active GIP, active SDF-1a

Mice were deeply anesthetized with sodium pentobarbital and transcardially perfused with cold saline. Brains were removed and midbrains with overlaying cortex dissected and snap frozen.

Determination of active GLP-1 (K150JWC-1, Mesoscale, Gaithersburg, USA) and active GIP (Immuno-Biological Laboratories, IBL 27724) was performed based on manufactures instructions. Active SDF-1α was detected as described by Fadini et al. [35].

### Liquid chromatography and mass spectrometry

Details on liquid chromatography and mass spectrometry are shown in Additional file 1.

### Statistics

Statistical analyses were performed using the unpaired t test with Welch’s correction (Study 1, 2), one-way ANOVA, followed by the Holm–Sidak’s multiple comparisons test (Study 3). For MS data receiver-operating characteristics (ROC) including the area under the curve (AUC) were calculated and corresponding p-values were determined using an unpaired t test with Welch’s correction (Study 4). Differences between groups were considered statistically significant when p < 0.05. Data are presented as mean ± SD.

## Results

### Sustained post-ischemic treatment with linagliptin improves motor function and reduces tissue damage after MCAO (Study 1)

**Fig. 1:**
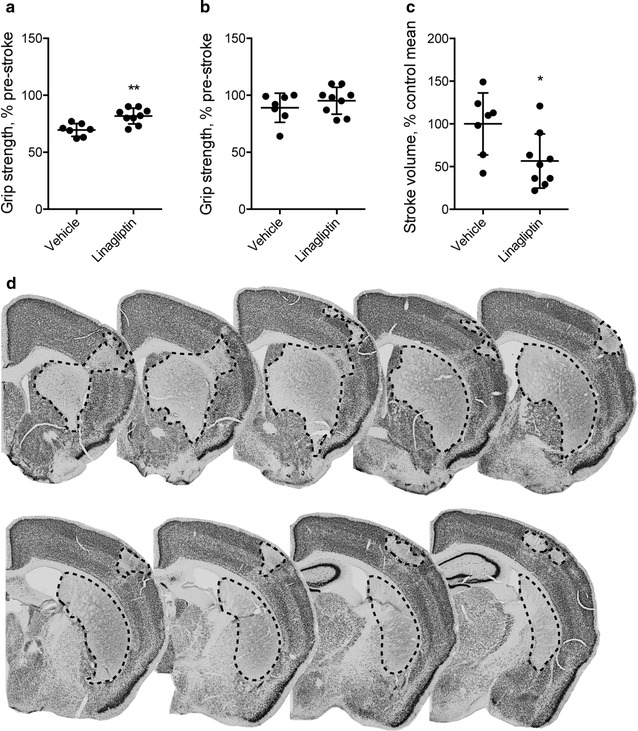
Effect of linagliptin treatment on motor function and infarct volume. **a** Forepaw grip strength at Day 3 and **b** 3 weeks after MCAO. **c** Infarct volume 3 weeks after MCAO. **d** Photomicrographs of NeuN immunoreactivity. Dashed line outlines the area of visible ischemic damage after MCAO. Unpaired t test with Welch’s correction, mean ± SD. * and ** denote p < 0.05 and p < 0.01 respectively

The effect of linagliptin treatment on motor function was evaluated 3 days and 3 weeks after MCAO. 3 days after MCAO, linagliptin-treated mice exhibited significantly smaller motor impairment in comparison to control mice (20% decrease in grip strength vs. 30% of that of vehicle group) (Fig. 1a). After 3 weeks, as expected [36], both groups had recovered motor performance to pre-stroke levels (Fig. 1b). The infarct volume (depicted in Fig. 1d) was evaluated 3 weeks after MCAO and showed a significant decrease (approximately 40% smaller) in linagliptin-treated mice (Fig. 1c).

### Treatment with linagliptin increases levels of active SDF-1a, but not of active GLP-1 and active GIP in the brain (Study 2)

**Fig. 2:**
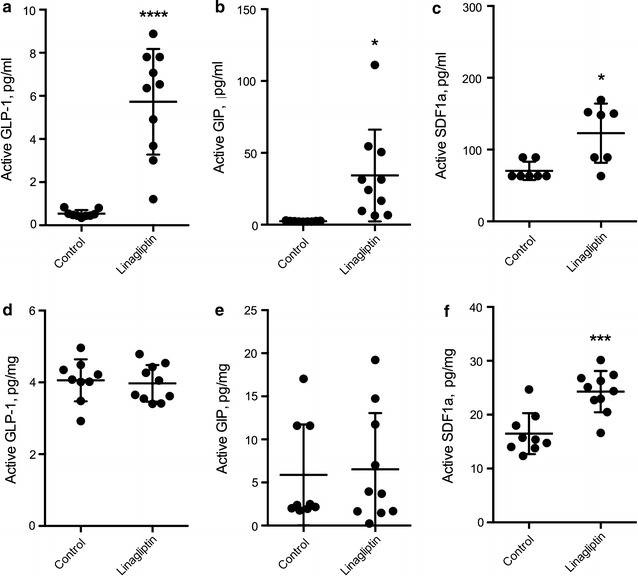
Effect of linagliptin treatment on active GLP-1, active GIP and active SDF-1a in serum and brain. Serum levels of active GLP-1 (**a**), active GIP (**b**) and active SDF-1α (**c**) after prolonged linagliptin treatment. Levels of active GLP-1 (**d**), active GIP (**e**) and active SDF-1α (**f**) in the brain after prolonged linagliptin treatment. Unpaired t test with Welch’s correction, mean ± SD. **, ***and **** denote p < 0.01, p < 0.001 and 0.0001 respectively

The results showed that sustained linagliptin treatment significantly increased levels of all three peptides in serum (Fig. 2a–c). However, the treatment had no effect on levels of active GLP-1 and active GIP (Fig. 2d, e respectively), but led to a significant increase (approximately 50%) of active SDF-1α (Fig. 2f) in brain homogenates.

### AMD3100 decreases positive linagliptin effects on motor function and tissue damage in the acute phase after stroke (Study 3)

**Fig. 3:**
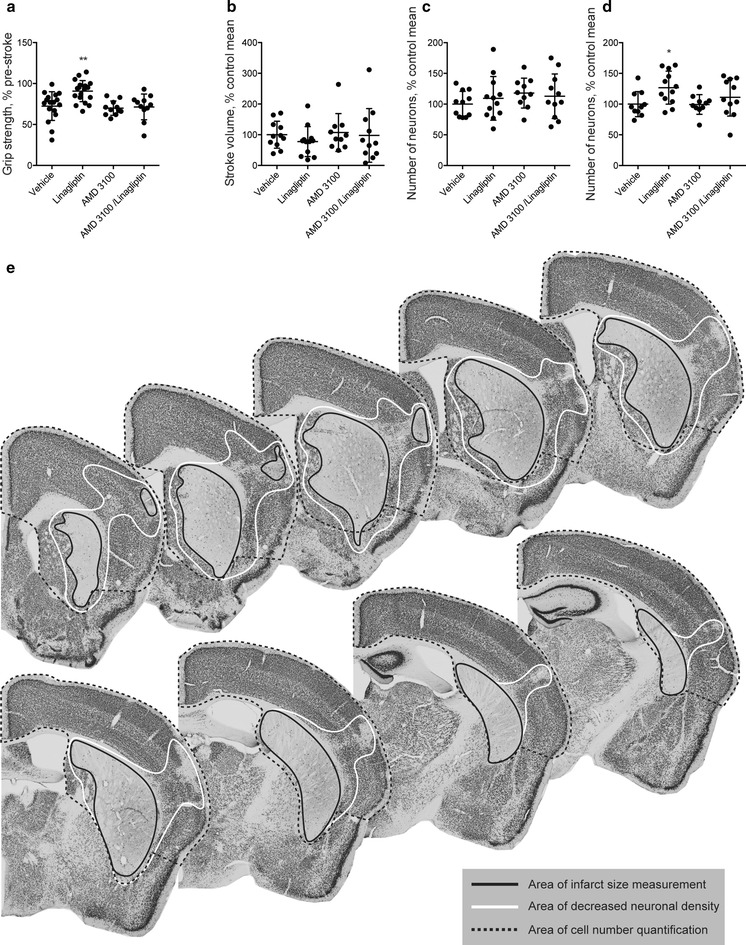
Effect of AMD3100 and linagliptin on motor function and tissue damage after MCAO. **a** Forepaw grip strength at Day 3 after MCAO. **b** Ischemic volume at Day 3 after MCAO. The number of surviving neurons in the striatum (**c**) and cortex (**d**) at Day 3 after MCAO. **e** Representative photomicrographs of brain sections (NeuN immunostained) used in the quantitative analyses. Solid black line denotes the area of the stroke infarct volume measurement. Solid white line denotes the area of decreased neuronal density after MCAO. Dashed black line denotes the area of neuronal quantification by stereology methods. One-Way ANOVA followed Holm–Sidak’s multiple comparisons test, mean ± SD. * and ** denote p < 0.05 and p < 0.01 respectively and indicate the statistically significant difference over the vehicle group

In Study 1 (see Fig. 1a), linagliptin showed a strong effect in improving upper-limb function at Day 3 after stroke. To test the potential involvement of the SDF-1α/CXCR4 pathway on this beneficial effect mediated by linagliptin, AMD3100 (specific antagonist of the SDF-1α/CXCR4) was administered from Day 1 before stroke until this time point. Moreover, the study was terminated at Day 3 after stroke to avoid the potential global side effects of chronic CXCR4 blocking. The results show that AMD3100 completely prevented the improvement of motor function induced by linagliptin (Fig. 3a). Moreover AMD3100 reduced the effect of linagliptin on histological outcome in the cortex (Fig. 3d). The neuroprotective effect of 3-days linagliptin treatment (based on histological analyses) was smaller as compared to 3-weeks treatment in Study 1 and was undetectable by ischemic volume measurements (Fig. 3b) or by quantifying surviving NeuN+ neurons in the striatum (Fig. 3c). This was probably due to a shorter linagliptin administration in comparison with Study 1 where the mice were treated for 3 weeks after stroke before sacrifice.

### Tandem mass spectrometry (Study 4)

We could not detect linagliptin in the brain under our experimental conditions.

47 differentially occurring peptides between groups from 16 different precursor proteins were identified (Table 1). Of these, the most frequent peptides originated from glyceraldehyde-3-phosphate dehydrogenase (G3P), neurogranin (NEUG) and an isoform of myelin basic protein (MBP).

**Table 1:** List of identified peptides

UniProt name	Protein name	Sequence	From–to
Higher signal intensities in LS and VS			
1433B_MOUSE	14-3-3 protein beta/alpha	L.WTSENQGDEGDAGEGEN.-	230–246
1433Z_MOUSE	14-3-3 protein zeta/delta	L.WTSDTQGDEAEAGEGGEN.-	228–245
RLA2_MOUSE	60S acidic ribosomal protein P2	-.MRYVASYLLAALGGN.S	1–15
ATPB_MOUSE	ATP synthase subunit beta, mitochondrial	A.AQASAAPKAGTATGRIVA.V	48–65
ATPB_MOUSE	ATP synthase subunit beta, mitochondrial	Y.AAQASAAPKAGTATGRIVA.V	47–65
ATP5H_MOUSE	ATP synthase subunit d, mitochondrial	M.AGRKLALKTIDWVSF.V + Acetyl (N-term)	2–16
KCRB_MOUSE	Creatine kinase B-type	L.IEMEQRLEQGQAIDDLMPAQK.-	361–381
IF4H_MOUSE	Eukaryotic translation initiation factor 4H	N.SAIFGGARPREEVVQKEQE.-	230–248
IF4H_MOUSE	Eukaryotic translation initiation factor 4H	M.ADFDTYDDRAYSS.F + acetyl (N-term)	2–14
G3P_MOUSE	Glyceraldehyde-3-phosphate dehydrogenase	N.RVVDLMAYMASKE.-	321–333
G3P_MOUSE	Glyceraldehyde-3-phosphate dehydrogenase	M.FQYDSTHGKFNGTVKAEN.G	45–62
G3P_MOUSE	Glyceraldehyde-3-phosphate dehydrogenase	L.ISWYDNEYGYSNRVVDLMAYMASKE.-	309–333
G3P_MOUSE	Glyceraldehyde-3-phosphate dehydrogenase	M.VKVGVNGFGRIGRLVTRA.A	2–19
G3P_MOUSE	Glyceraldehyde-3-phosphate dehydrogenase	M.VKVGVNGFGRIGRLVT.R	2–17
GBB2_MOUSE	Guanine nucleotide-binding protein G(I)/G(S)/G(T) subunit beta-2	M.SELEQLRQEAEQLRN.Q + acetyl (N-term)	2–16
ESTD_MOUSE	S-Formylglutathione hydrolase	T.FIADHIRHHAKYLNA.-	268–282
TPIS_MOUSE	Triosephosphate isomerase	M.APTRKFFVGGN.W	52–62
G6PI_MOUSE	Glucose-6-phosphate isomerase	M.AALTRNPQFQKLLEWHRAN.S + acetyl (N-term)	2–20
G6PI_MOUSE	Glucose-6-phosphate isomerase	N.GLISFIKQQRDTKLE.-	544–558
Higher signal intensities in VS, LC, VC			
DPYL2_MOUSE	Dihydropyrimidinase-related protein 2	S.SAKTSPAKQQAPPVRNLH.Q	518–535
DPYL2_MOUSE	Dihydropyrimidinase-related protein 2	V.APPGGRANITS.L	560–570
DPYL2_MOUSE	Dihydropyrimidinase-related protein 2	V.APPGGRANITSLG.-	560–572
EAA2_MOUSE	Excitatory amino acid transporter 2	M.ASTEGANNMPKQVEVRMHDSHLS.S + acetyl (N-term)	2–24
EAA2_MOUSE	Excitatory amino acid transporter 2	M.ASTEGANNMPKQVEVRMHDSH.L + acetyl (N-term)	2–22
MBP_MOUSE	Myelin basic protein	V.TPRTPPPSQGKG.R	93–104
MBP_MOUSE	Myelin basic protein	R.TPPPSQGKGRGLS.L	96–108
MBP_MOUSE	Myelin basic protein	I.VTPRTPPPSQGKG.R	92–104
MBP_MOUSE	Myelin basic protein	N.IVTPRTPPPSQGKGRGLSLS.R	91–110
MBP_MOUSE	Myelin basic protein	N.IVTPRTPPPSQGKG.R	91–104
MBP_MOUSE	Myelin basic protein	R.TPPPSQGKGRGLSLS.R	96–110
MBP_MOUSE	Myelin basic protein	V.TPRTPPPSQGKGRGLSLS.R	93–110
MBP_MOUSE	Myelin basic protein	K.RPSQRSKYLATA.S	6–17
MBP_MOUSE	Myelin basic protein	Q.KRPSQRSKYLATA.S	5–17
MBP_MOUSE	Myelin basic protein	M.ASQKRPSQRSKYLAT.A + acetyl (N-term)	2–16
MBP_MOUSE	Myelin basic protein	M.ASQKRPSQRSKYLATA.S + acetyl (N-term)	2–17
MBP_MOUSE	Myelin basic protein	M.ASQKRPSQRSKYLATAS.T + acetyl (N-term)	2–18
MBP_MOUSE	Myelin basic protein	M.ASQKRPSQRSKYLATAST.M + acetyl (N-term)	2–19
MBP_MOUSE	Myelin basic protein	M.ASQKRPSQRSKYLATASTMD.H + acetyl (N-term); oxidation (M)	2–21
MBP_MOUSE	Myelin basic protein	M.ASQKRPSQRSKYLATASTMDH.A + acetyl (N-term); oxidation (M)	2–22
MBP_MOUSE	Myelin basic protein	M.ASQKRPSQRSKYLATASTMDHA.R + acetyl (N-term)	2–23
MAG_MOUSE	Myelin-associated glycoprotein	G.KRPTKDSYTLTEELAEY.A	604–620
NEUG_MOUSE	Neurogranin	K.GPGPGGPGGAGGARG.G	55–69
NEUG_MOUSE	Neurogranin	P.GGPGGAGGARGGAGGGPSGD.-	59–78
NEUG_MOUSE	Neurogranin	R.KGPGPGGPGGAGGARGGAGGGP.S	54–75
NEUG_MOUSE	Neurogranin	K.GPGPGGPGGAGGARGGAGGGPSGD.-	55–78
NEUG_MOUSE	Neurogranin	R.KGPGPGGPGGAGGARGGAGGGPSGD.-	54–78
NEUG_MOUSE	Neurogranin	G.RKGPGPGGPGGAGGARGGAGGGPSGD.-	53–78
SNG3_MOUSE	Synaptogyrin-3	A.YPGYPVGSGVEGTETY.Q	193–208

The table depicts the UniProt name, the precursor protein name, the amino acid sequence and the amino acid range of identified peptides. The dot within the sequence denotes the cleavage site. The top part list peptides found to possess significant higher signal intensities in stroke samples and the bottom part peptides possessing significant lower signal intensities in linagliptin-stroke samples

G3P is a key cytosol enzyme in glycolysis. Linagliptin stroke (LS) and vehicle stroke (VS) groups showed higher signal intensities of 5 unique G3P-derived peptides in comparison to linagliptin non-stroke (LC) and vehicle non-stroke (VC) groups (Table 1).

**Fig. 4:**
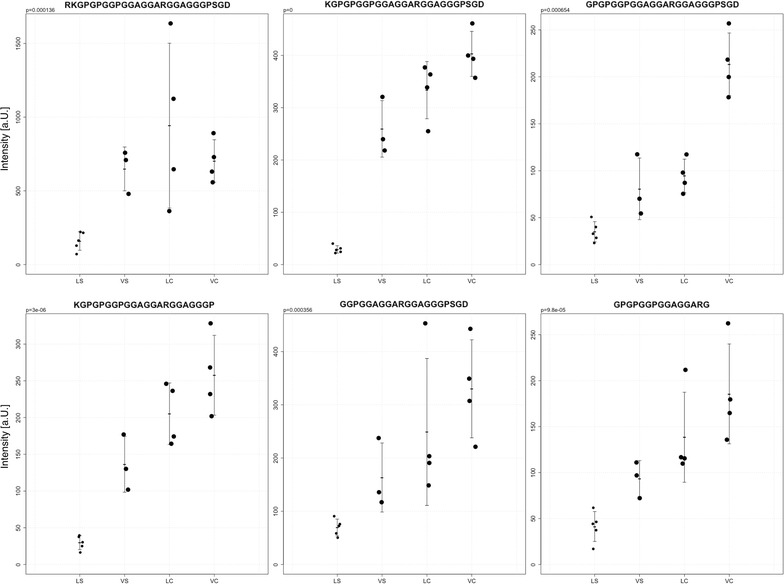
Detection of neurogranin peptides. The figure shows signal intensities, means and SD of 6 neurogranin (NEUG)-derived peptides (NEUG 53–78, NEUG 54–78, NEUG 55–78, NEUG 54–75, NEUG 59–78 and NEUG 55–69) in 16 brain samples (*LS* linagliptin-stroke, *VS* vehicle stroke, *LC* linagliptin control, *VC* vehicle control). The star marks significant differences (ROC-AUC = 1, p < 0.005) between LS and the remainder of samples. The corresponding amino acid sequence is depicted at the top of each graph

NEUG is primarily expressed in the brain [37] and is the main postsynaptic protein regulating the availability of Calmodulin by binding to it in the absence of Ca^2+^ [38]. Here, signal intensities of 6 unique peptides were lower in LS in comparison to the remainder of samples (VS, LC, VC) (Fig. 4).

**Fig. 5:**
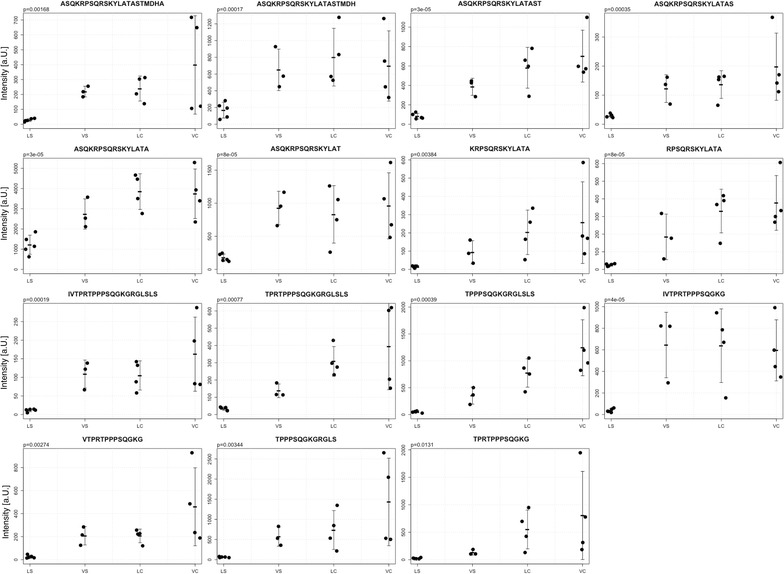
Detection of myelin-basic protein (MBP). The figure shows signal intensities of peptides with means and standard deviations in each group (*LS* linagliptin-stroke, *VS* vehicle stroke, *LC* linagliptin control, *VC* vehicle control) derived an isoform of myelin basic protein (MBP). The p-value was calculated between LS and the remainder of samples. In all cases the ROC-AUC was 1 for LS

MBP is one of the most abundant protein components of the myelin membrane in the CNS [39] which also binds Calmodulin [40]. LS showed lower signal intensities of 15 unique peptides in comparison to the remainder of samples (VS, LC, VC), (Fig. 5).

## Discussion

The primary objective of this study was to determine whether the improved outcome after stroke following gliptin treatment is SDF-1α/CXCR4-dependent. We showed that linagliptin improves functional stroke outcome in a SDF-1α/CXCR4-dependent manner. Secondarily, we demonstrated that linagliptin after stroke decreased the presence of peptides derived from NEUG and MBP.

Different research groups have shown that gliptins reduce brain damage and improve functional parameters after stroke in various animal models independently from a T2D background (reviewed in [2, 41, 42]). A few large clinical studies with gliptins in diabetic patients have investigated the potential of these drugs to decrease cardiovascular incidence (including stroke) and death with neutral results (reviewed by Nauck et al. [10]). However, since the efficacy measures in these clinical studies (stroke incidence and death) did not address functional outcomes after stroke, further clinical studies are needed to evaluate the potential of these drugs to improve functional stroke outcome [9]. Interestingly, the ongoing CARMELINA study with linagliptin (Clinicaltrials.gov; NCT01897532) contains a post-stroke functional sub-study using the modified Rankin scale to assess stroke-induced disability approximately 1 week following stroke and at ~ 3 months after stroke-onset. Preclinical data indicating that gliptins can improve stroke outcome in the post-stroke recovery phase have been recently shown by Ma et al. in a model of transient cerebral ischemia induced by bilateral common carotid artery occlusion. The study showed that sustained linagliptin treatment after cerebral ischemia counteracted cognitive impairment and brain atrophy, independently from the regulation of glycemia [43]. This study is remarkable because their model allows extending the observation period for several weeks after artery occlusion thus evaluating effects of sustained gliptin treatment in the post-stroke recovery phase. Our results confirm that sustained linagliptin treatment after stroke is necessary to improve stroke outcome while a single acute bolus administration of linagliptin at stroke time was ineffective (previously published in [13]).

Clinical data suggesting that gliptins can exert beneficial effects in the damaged brain do also exist. Isik et al. recently showed that a treatment for 6 months with sitagliptin was associated with improvement of cognitive function in elderly diabetic patients with and without Alzheimer’s disease [8]. To support the translation of preclinical functional outcome stroke studies with gliptins to clinical settings, it is helpful to identify mechanisms of action of this class of drugs in the brain.

The antidiabetic effects of gliptins are mediated via GIP and GLP-1 regulation, but other incretin-independent mechanisms may also be involved [15]. We recently showed that mice lacking the GLP-1-receptor exhibit improved stroke outcome after linagliptin treatment [13]. Recent research by Han et al. has shown that a dual agonist targeting both GLP-1 and GIP receptors promoted stronger neuroprotection against stroke than the GLP-1 analogue Val(8)-GLP-1(glu-PAL) alone thus suggesting a mechanism mediated by GIPR activation [14]. Further studies employing mice lacking GIP and/or GIPR are needed to investigate this hypothesis. However, we showed in this study that brain GIP levels were unaffected by linagliptin treatment. Furthermore our previous study using mice lacking the GLP-1R [13] showed that linagliptin can improve stroke outcome independently from GLP-1R. Although we cannot rule out peripheral effects mediated by GIP, this suggests that the positive effect of gliptins on stroke outcome may not be necessarily related to incretins.

The DPP-4 substrate SDF-1α plays a pivotal role in the brain, as it regulates neurovascular remodeling after stroke [23–26]. The beneficial effects of SDF-1α were also shown in rats after traumatic brain injury [44] and in an AD animal model, where SDF-1α treatment decreased beta-amyloid deposition [45]. Of relevance for our study, recent research in myocardial infarction (MI) has shown that increased SDF-1α by gliptins mediates protective effects against MI through anti-apoptotic effects [46, 47].

Our results show that sustained linagliptin treatment increases active SDF-1α in brain parenchyma. Importantly, by sustained blocking of the SDF-1α/CXCR4 pathway, linagliptin-mediated effects on functional and histological outcomes after stroke were diminished. This indicates that improved stroke outcome by linagliptin occurs via the activation of the SDF-1α/CXCR4 pathway. DPP-4 activity was similarly inhibited in linagliptin, and linagliptin/AMD 3100-treated animals (data not shown). Therefore, the inhibitory effect of AMD 3100 over linagliptin on stroke outcome could not be linked to altered DPP-4 activity between the groups. The pro *versus* adverse effects of SDF-1α after gliptins treatments in diabetic complications has been recently deeply discussed [48, 49]. Our results suggest that, at least when it comes to post-stroke treatment, the activation of the SDF-1α/CXCR4 pathway promotes beneficial effects. Two weaknesses of this set of results that need to be addressed in the future are: (1) the fact that the study was performed in naïve mice and that a diabetic background could have affected the outcome; (2) it is unclear why the SDF-1α/CXCR4 pathway seems to be more involved in the functional (Fig. 3a) rather than in the structural recovery (Fig. 3d). Nevertheless, previous studies show that SDF-1α is involved in axonal path finding, outgrowth and branching; all functions involved in functional recovery [19].

The effects of linagliptin to reduce the injury after stroke could involve the neuroprotective, non-neurogenic rapid effects of neural progenitor cells (NPCs) [50] since CXCR4 inhibition in NPCs leads to failure of newborn neurons to localize to the ischemic brain tissue [19]. The linagliptin effects via SDF-1α/CXCR4 to reduce the brain injury after stroke could also be mediated by the regulation of neovascularization through endothelial progenitor cells (EPCs) [51] since gliptins increase ischemic angiogenesis by preserving EPCs function [52]. Moreover, SDF-1α is known to be involved in the recruitment to the injury region of EPCs [19]. This action of SDF-1α on EPCs could contribute to explain the stronger efficacy of linagliptin to improve stroke outcome after 3 weeks versus 3 days of treatment as it has been shown that EPCs recruitment occurs 2 weeks after ischemic injury [53]. New studies should be performed in the future to demonstrate this hypothesis.

Finally, the SDF-1α/CXCR4 pathway could play a role in functional regulation of the brain vasculature, since linagliptin improves endothelium-dependent relaxation independently of glucose regulation [28]. Furthermore, SDF-1α/CXCR4 signaling activates endothelial nitric oxide synthase [32] which is a key enzyme maintaining homeostasis by inducing vasodilatation and whose impairment is implicated in the pathogenesis of stroke [54]. These results support the possibility that the positive effects of gliptins on stroke outcome could also occur via increased blood perfusion in collateral vessels in the *penumbra* region of ischemic brains thus mitigating the degenerative effects of MCAO.

To further elucidate effects of linagliptin after stroke, we analyzed brain tissue samples by mass spectrometry. We did not detect linagliptin under our experimental conditions in agreement with previous published data demonstrating that linagliptin does not cross the blood brain barrier under physiological conditions [55]. However due to MALDI-TOF/TOF mass spectrometry conditions, we cannot rule out that traces of linagliptin could enter the brain under stroke conditions.

Peptides that are generated by proteolytic processing of larger precursor proteins can be regarded as surrogate markers for expression levels of proteins or peptidase activity [56]. In samples from animals subjected to MCAO, peptides derived from G3P were observed. We speculate that these peptides reflect a direct effect of ischemia causing release of substances from the cytosol due to cell membrane instability.

The analysis further revealed that NEUG peptides exhibit lower signal intensities in stroke tissue samples from animals treated with linagliptin (Fig. 4 and Figure S2 in Additional file 1). These results might suggest lowered expression of NEUG or a reduced susceptibility to proteolytic processing of free NEUG not bound to Calmodulin.

The protein level or proteolytic cleavage pattern of an isoform of MBP appeared to be also altered (Fig. 5). The observed pattern of MBP peptides (Figure S3 in Additional file 1) shows a very good fit to Calpain processing based on the substrate and observed cleavage sites according to MEROPS, a database of proteolytic enzymes [57]. We presume that the observed MBP peptides do not represent breakdown products due to stroke since no significant difference between VS and LC/VC samples was present. Rather the low intensities or even diminished presence of MBP peptides in samples from mice subjected to stroke and treated with linagliptin, mirror altered proteolytic activities or insusceptibility to processing of MBP due to e.g. Calmodulin binding [40] as a second-tier effect (see below) analog to NEUG. These peptides are not affected in samples from linagliptin-treated control mice, which is in line with the observation that linagliptin does not cross the blood brain barrier under physiological conditions [55].

These data suggest that the proteolytic processing products of two Calmodulin-binding proteins exhibit significantly lower signal intensities in brain samples after stroke under linagliptin treatment either due to altered expression, insusceptibility to proteolytic processing because of Calmodulin binding and/or altered activity of proteases like Calpain. Since presence of free NEUG and MBP and/or altered Calpain activity are all dependent on intracellular Ca^2+^ concentration [38, 58], we hypothesize that linagliptin treatment, presumably through SDF-1α, could affect Ca^2+^ homeostasis. Indeed, a study by Nicolai et al. [59], showed that SDF-1α selectively inhibits the expression of NR2B, a regulatory subunit of NMDA receptor, altering NMDA-induced Ca^2+^ responses associated with neuronal death, while promoting pro-survival pathways. However further studies (intracellular Ca^2+^ measurement, Calpain activity) are necessary to verify or falsify the proposed mechanism.

## Conclusions

T2D patients have more than double the risk of ischaemic stroke [60]. The surviving stroke patients with T2D often show poor functional recovery and even further neurological deterioration in comparison to non-diabetics. This holds true even after adjusting for stroke severity and patients’ age [61]. Remarkably, this important problem has been poorly investigated clinically in comparison to several studies focused on why the incidence of stroke in T2D patients is higher than in non-diabetics. Therefore, a treatment with gliptins in the acute phase after stroke could be beneficial for T2D patients based on preclinical studies. However, more research on the mechanisms at the basis of gliptins action is needed to exploit their potential properties. We did demonstrate the involvement of the SDF-1α/CXCR4 pathway in improved stroke outcomes after linagliptin treatment. Our data also suggest a potential gliptin-mediated neuroprotective mechanism that involves NEUG and MBP through the regulation of Ca^2+^ homeostasis and the reduction of Calpain activity. Although these results provide a first glance of gliptin actions in the brain only, they represent new insights on effects of these anti-hyperglycemic drugs against decreased functional outcome after stroke.

## Additional file

**Additional file 1.** Additional methodological information and 2 additional results figures.
