# Supplementary material for: The effect of DPP-4 inhibition to improve functional outcome after stroke is mediated by the SDF-1α/CXCR4 pathway
Source: Cardiovasc Diabetol. 2018 May 19;17:60. doi: 10.1186/s12933-018-0702-3 (PMC5960142; doi:10.1186/s12933-018-0702-3)
Supplement: Supplementary file 1 — Additional file 1. Additional methodological information and 2 additional results figures. [file 12933_2018_702_MOESM1_ESM.docx]

**Additional information**

**Materials and Methods**

***Experimental design***

***
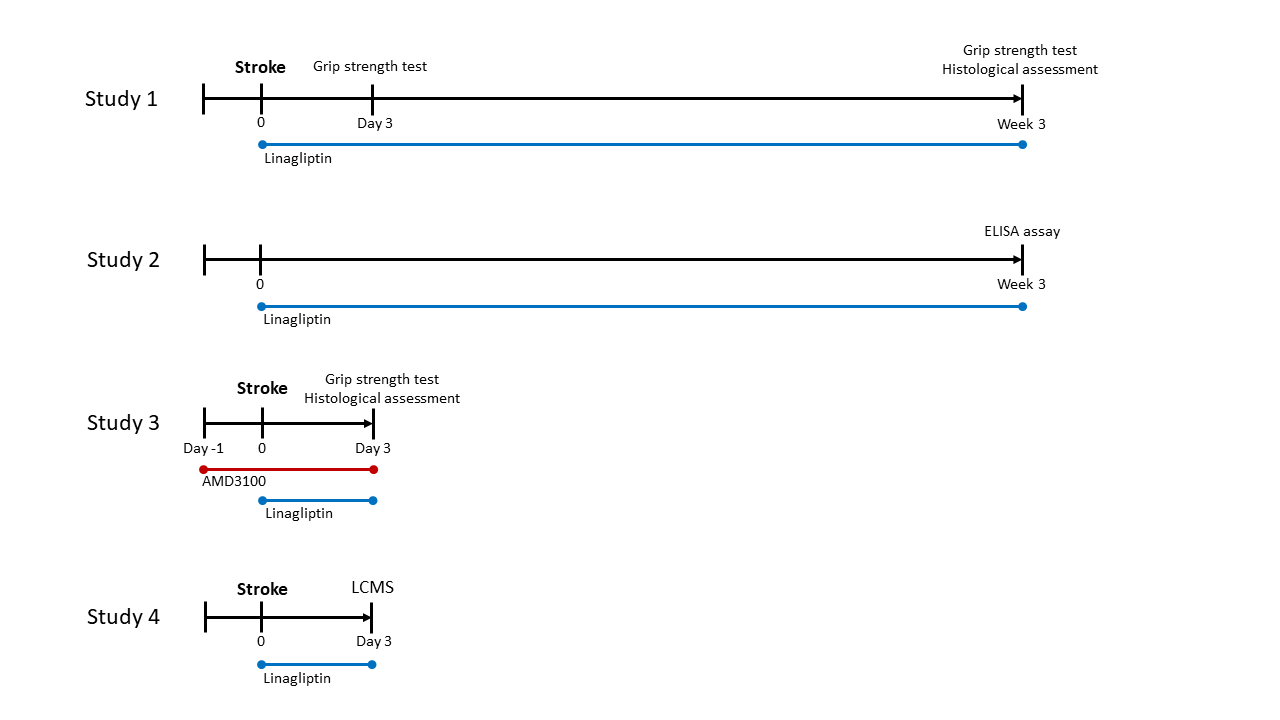
***

**Figure S1**

Representative illustration of study design.

***Immunohistochemistry, infarct volume and cell quantifications***

The primary antibody anti-NeuN (1:100; Millipore, MA) was used to stain surviving neurons in striatum and cerebral cortex. Sections were incubated with the primary antibody for 36 hours at 4°C in phosphate buffer containing 3% normal horse serum and 0.25% Triton-X. Primary antibodies were detected using biotin-conjugated anti mouse (Vector, CA) secondary antibodies (1:200). Sections were incubated with secondary antibodies for 2 hours at room temperature in phosphate buffer containing 3% normal horse serum and 0.25% Triton-X. For chromogenic visualization, avidin-biotin complex (ABC kit, Vector, CA) and diaminobenzidine were used.

An investigator blinded to the experimental groups performed both tissue damage quantification and cell counting. For tissue damage evaluation, the NeuN-labeled tissue sections were displayed live on the computer monitor and the area of contralateral hemisphere and the area of the intact ipsilateral tissue were measured in every section containing stroke damage using NewCast (Visiopharm, Hoersholm, Denmark) software. To compensate for the stroke-induced morphological tissue changes, the infarct volume was calculated by subtracting the volume of remaining intact tissue in the ipsilateral hemisphere from the volume of the contralateral hemisphere. The values from vehicle group were used to calculate the average size of ischemic damage without the drug treatment and assigned a value of 100%. The values of ischemic volume from all individual animals were then normalized against the vehicle average (100%).

Immunoreactive cells were counted in striatum and cortex by using a computerized non-biased setup for stereology, driven by NewCast software. The number of neurons were quantified using the optical fractionator method [1, 2]. Briefly, brain sections were displayed live on the computer monitor and the striatum and cortex delineated at low magnification. Quantifications were performed using a 40x dry lens with numeric aperture of 1.30 and 2x digital zoom. Nine evenly spaced sections in parallel-cut series through the entire striatum were included. Random sampling was carried out using the counting frame, which systematically was moved at predefined intervals so that approximately 300 immunoreactive cells were counted. The total number of cells was estimated according to the optical fractionator formula [1, 2]. The values from vehicle group were used to calculate the average number of surviving neurons without the drug treatment and assigned the value of 100%. The cell numbers from all individual animals were then normalized against the vehicle average (100%).

***Liquid chromatography and mass spectrometry***

16 brain homogenates from four groups (5 Linagliptin-stroke (LS) 3 vehicle stroke (VS), 4 Linagliptin control (LC), 4 vehicle control (VC)) were separated by liquid chromatography into 96 fractions and subsequently subjected to mass spectrometric analysis [3] Briefly, after peptide extraction 6 mg equivalent of brain tissue (n=16) was separated by reversed phase liquid chromatography into 96 fractions. After separation, each fraction was subjected to matrix-assisted laser desorption ionization time of flight (MALDI-TOF) mass spectrometry (4700 Analyzer, Applied Biosystems, Framingham, USA) in linear mode. After mass spectrometric data acquisition, spectra were analyzed, including peak recognition and visualization using the software package Spectromania developed in-house and R [4] including the MALDIquant package [5]. In total 1536 mass spectra were generated and analyzed by univariate statistics to reveal differentiating signals between groups. To conserve native proteolytic cleavage sites no tryptic digestion was performed. Peptide identification was achieved by MALDI-TOF/TOF mass spectrometry (5800 Analyzer, Applied Biosystems, Framingham, USA). Tandem mass spectrometry data was subsequently noise filtered and peak deisotoped and saved in Mascot (Matrix Science, London, UK) generic file format and submitted to the Mascot search engine. Cascading searches in UNIPROT (version 2017_03, www.uniprot.org), including common post-translational modifications, were performed.

**

 Results**

**Figure S2**

The figure shows the detection of Neurogranin (NEUG)-derived peptides in 16 brain samples (LS: Linagliptin-Stroke, VS: Vehicle Stroke, LC: Linagliptin Control, VC: Vehicle Control). A: Mass spectrometric data from one chromatographic fraction in a mass range between 1300 and 2100 Dalton. Each lane represents the mass spectrometric data from the same chromatographic fraction from each sample within the specified mass range. NEUG peptides are marked (1-3). B: Mean mass spectrometric spectra calculated from individual spectra from each group. The small numerals below each peak refer to the mean signal to noise ratio of a given signal.





**Figure S3**

The figure depicts the amino acid sequence of the precursor protein with initiator Methionine corresponding to isoform 3 of Myelin basic protein. The underlined sequence indicates sections where all identified peptides derived from. The dot indicates the acetylated Alanine. Since Alanine at position 2 was found to be acetylated this indicates that identified peptides represent the N-terminus of the expressed protein and therefore identified MBP-peptides most likely correspond to isoforms 4, 5, 6 or 8. Below the number of identified peptides is depicted. Additionally, the alignment of sequenced peptides is shown. These peptides derived from two regions of the precursor protein and exhibit a diverse set of N -and C-terminal cleavage sites indicating a processing by presumably Calpain.

**References**

[1] West MJ, Slomianka L, Gundersen HJ. Unbiased stereological estimation of the total number of neurons in thesubdivisions of the rat hippocampus using the optical fractionator. Anat Rec. 1991; **231**: 482-497

[2] West MJ. Stereological methods for estimating the total number of neurons and synapses: issues of precision and bias. Trends in neurosciences. 1999; **22**: 51-61

[3] Tammen H, Hess R, Rose H, Wienen W, Jost M. Peptidomic analysis of blood plasma after in vivo treatment with protease inhibitors--a proof of concept study. Peptides. 2008; **29**: 2188-2195

[4] Team RC. R: A Language and Environment for Statistical Computing. Vienna, Austria: R Foundation for Statistical Computing; . 2015:

[5] Gibb S, Strimmer K. MALDIquant: a versatile R package for the analysis of mass spectrometry data. Bioinformatics. 2012; **28**: 2270-2271
